# The emerging role of circular RNAs in spinal cord injury^☆^

**DOI:** 10.1016/j.jot.2021.06.001

**Published:** 2021-07-22

**Authors:** Peng Peng, Hao Yu, Yongjin Li, Jingyuan Huang, Shengyu Yao, Cong Xing, Weixiao Liu, Bin Zhang, Shiqing Feng

**Affiliations:** a Department of Orthopedics, Tianjin Medical University General Hospital, No.154 Anshan Road, Heping District, Tianjin, 300052, China; b International Science and Technology Cooperation Base of Spinal Cord Injury, Tianjin Key Laboratory of Spine and Spinal Cord Injury,Department of Orthopedics, Tianjin Medical University General Hospital, Tianjin, China; c Department of Orthopedics, Kuancheng Manzu Autonomous Country Hospital, Chengde, China

**Keywords:** CircRNAs, Spinal cord injury, Nerve regeneration

## Abstract

Spinal cord injury (SCI) is one kind of severe diseases with high mortality and morbidity worldwide, and lacks effective therapeutic interventions currently, which leads to not only permanent neurological impairments but also heavy social and economic burden. Recent studies have proved that circRNAs are highly expressed in neural tissues, regulating the neuronal and synaptic functions. What's more, significantly altered circRNAs expression profiles are closely associated with the pathophysiology of SCI. In this review, we summarize the current advance on the role of circRNAs in SCI, which may provide a better understanding of pathogenesis and therapeutic strategies of SCI.

**The Translational potential of this article:**

The Translational potential of this article is that A further understanding of circRNAs in the pathogenesis of SCI will promote the circRNA-based clinical applications.

## 1 Introduction

Spinal cord injury (SCI) is one kind of severe diseases with high mortality and morbidity worldwide, and lacks effective therapeutic interventions currently, leading to not only permanent neurological impairments but also heavy social and economic burden [[1], [2], [3]]. What's more, SCI undergoes primary injury and secondary neurological damage, which involves common pathophysiological mechanisms such as inflammation, autophagy, blood–brain barrier disruption, and neuronal apoptosis [[4], [5], [6], [7]]. The complex biological processes under SCI and the role of specific molecules in nerve regeneration need further research.

More than 98% of transcriptional output is composed of non-coding RNAs (ncRNAs), which could regulate transcription, translation, epigenetic modification and various biological processes or diseases [[8], [9], [10]]. Circular RNAs (circRNAs), one emerging kind of ncRNAs with covalently closed loop structures formed by back splicing, exhibit high abundance, stability and tissue/stage specificity in the central nervous system (CNS) [[11], [12], [13], [14]]. Recently, accumulating evidence indicates that circRNAs expression profiles are significantly altered following acute CNS injuries, suggesting the close associations of circRNAs with the pathophysiology of SCI [[15], [16], [17], [18]]. This review highlights the characteristics and regulatory role of circRNAs in SCI, facilitating a further understanding of the pathogenesis of SCI and promoting the circRNA-based clinical applications.

## 2 Summary of circRNAs

### 2.1 Origins, characteristics and classifications of circRNAs

In recent years, circular RNAs (circRNAs) are an emerging class of ncRNAs and have become a research hotspot in the RNAs field. Contrary to common linear RNAs, circRNAs are formed by a covalently linked ends without 5′ caps and 3’ poly-A tails [19,20]. Based on this unique circular structure, circRNAs show resistance to exonucleolytic degradation caused by RNase R or/and RNA exonuclease, which accounts for the high abundance and stability in mammalian cells [[21], [22], [23]].

CircRNAs can be mainly divided into three types: exonic circRNAs (ecircRNAs), intronic RNAs (ciRNAs) and exon-intron circRNAs (EIciRNAs). EcircRNAs are derived only from exons and represent the largest group of circRNA classes. They are located predominately in the cytoplasm and might function as miRNAs sponges, regulate parental genes transcription and protein functions. Different from ecircRNAs, ciRNAs, a small fraction of circRNAs, are exclusively consisted of introns (including intron lariats) and accumulated in the nucleus where regulate their parental mRNAs at both the transcription and splicing levels. As a new subclass of circRNAs, EIciRNAs contain at least two exons and one retained intron. Like ciRNAs, EIciRNAs also display nuclear localizations and functions of regulating gene transcription [13,[24], [25], [26], [27]].

The majority of circRNAs are generated by back splicing, which includes intron pairing-driven circularization, RNA binding proteins (RBPs) pairing driven circularization and exon skipping [[28], [29], [30]]. For example, ecircRNA and EIciRNAs biogenesis are dependent on a combinatorial manner of long flanking introns (such as repetitive Alu pairs), specific RBPs (such as Quaking I (QKI) and Muscle blind (MBL)) and the lariat-containing exons [[31], [32], [33], [34]]. Besides, the process of ciRNAs formation relies on a consensus motif featured by a 7-nt GU-rich element approaching the 5′ splice site and an 11-nt C-rich element near the branch site [29,35].

### 2.2 The regulatory roles of circRNAs

As numerous studies have pointed out that circRNAs take part in not only extensive life processes but also various human diseases pathogenesis. It has been suggested that circRNAs could act as miRNAs sponges, regulate gene transcription, and even could be translated into proteins [[36], [37], [38]].

The competitive endogenous RNAs (ceRNA) hypothesis proposes that circRNAs contain shared miRNA response elements (MREs) to competitively bind with miRNAs, thereby counteracting miRNA silencing effect [[39], [40], [41]]. Cerebellar degeneration-related protein 1 antisense RNA (CDR1as), also known as ciRS-7, contains over 70 binding sites for miR-7 to act as a potential miR-7 sponge in cancers, diabetes as well as neural development and diseases, including Parkinson's disease (PD), Alzheimer's disease (AD) and neuropsychiatric disorders [[42], [43], [44], [45]]. As one of the most widely studied circRNAs in the CNS, ciRS-7 is significantly downregulated in the hippocampus of sporadic AD patients [46]. Moreover, ectopic expression of ciRS-7 causes a specific reduction of midbrain size in the zebrafish [44]. Although the role of miR7 in repressing α-synuclein in PD and regulating dendritic spine density in neuropsychiatric disorders was identified, whether it is ciRS-7-miR7 interactions works or not remains to be determined [45,47].

Besides ciRS-7, circular RNA sex-determining region Y (circular SRY) could serve as a miR-138 sponge by binding with16 putative target sites [44]. Circ-HIPK3, formed from Exon2 of the HIPK3 gene, acts as a sponge for multiple miRNAs to regulate human cell proliferation [48]. What's more, circular RNA itchy E3 ubiquitin protein ligase (circular ITCH) could compete with miR-7 and miR-214 to play an anti-tumor role in colorectal cancer and lung cancer proliferation by regulating the Wnt/β-catenin pathway [49,50].

In regard to gene transcription, the back splicing of circRNAs may exhibit competitive inhibition with canonical splicing of pre-mRNAs to result in lower levels of linear mRNA expression. However, it's also worth noting that some nuclear ciRNAs and EIciRNAs, such as ci-ankrd52, circSEP3, circEIF3J and circPAIP2 RNA, could enhance gene transcription mediated by polymerase II (Pol II) and U_1_ small nuclear ribonucleoprotein particle (U_1_snRNP) complex [31,51,52]. Therefore, the final gene expression levels are not determined only by some specific circRNAs since a single target may have multiple MREs and regulating mechanisms.

Due to the fact that circular RNAs contain coding exons and carry open reading frames, circRNAs may have the ability to produce proteins. Recent studies demonstrate that circRNAs translations can be efficiently driven using short sequences containing N_6_-methyladenosine (m6A) site as internal ribosome entry sites (IRESs) [53,54]. Human circ-ZNF609 containing a 753-nt open reading frame (ORF) was observed to modulate myoblast differentiation via encoding corresponding protein [55]. Although many circRNAs may have translation potential, their functional significance needs to be further identified.

### 2.3 CircRNAs and central nervous system

Accumulating evidence has shown that circRNAs are significantly enriched in CNS, more interestingly, exhibiting tissue/cell- and developmental-stage-specific expression patterns [14]. During neuronal differentiation, the expression of circRNAs is increased and thousands of circRNAs are significantly enriched in synapses, suggesting potential roles of circRNAs in neurogenesis. What's more, using high resolution in situ hybridization, the expression changes of circRNAs are directly visualized in the dendrites of neurons during neuronal development and neural plasticity, indicating a dynamic response to synaptic function [56].

Except for the synaptic genes, a part of synaptically-enriched circRNAs derived from the genes that participate in various biological processes including the transforming growth factor (TGF-β) pathway, axon guidance and Wnt signaling pathway [15,57,58]. Furthermore, Yang et al. [59] investigated circRNAs expression profiles of mouse neural stem cells during the proliferation and differentiation, which revealed complex circRNA-mRNA modulated mechanisms. Together, all these studies demonstrate the abundance of circRNAs in mammalian brains and potential neuronal regulatory functions.

## 3 CircRNAs in SCI

Spinal cord injury (SCI), another kind of neurotrauma, is a devastating and complex disease with approximately 250,000–500,000 people affected each year, which causes permanent neurological deficits [60,61]. Recently, increasing studies have demonstrated that circRNAs play an important role in the pathogenesis of SCI and they may be the potential therapeutic targets. To investigate the potential mechanism and search for therapeutic targets, several studies about circRNAs expression profiles in SCI have been reported, indicating the important regulatory roles of circRNAs in SCI (Table 1).

**Table 1:** List of studies on circRNAs alteration after SCI.

SCI model	Injury sites	Time points	Methods	CircRNAs expression profiles	Reference
Rat contusion SCI	T10	2 ​h	Microarray	1101 up	Liu et al. [62]
				897 down	
Rat contusion SCI	T9	6 ​h	RNA-Seq	99 up	Zhou et al. [63]
				51down	
Rat contusion SCI	T8	24 ​h	qRT-PCR	CircRNA_ 0001723	Li et al. [79]
Rat contusion SCI	T10	3 ​d	Microarray	415 up	Qin et al. [67]
				1251 down	
Rat contusion SCI	T10	3 ​d	Bioinformatic analysis	4 core DEcircRNAs	Peng et al. [71]
Mice contusion SCI	T9	3 ​d	RNA-Seq	249 up	Wang et al. [72]
				249down	
Mice contusion SCI	T10	3 ​d	Microarray	909 up	Yao et al. [73]
				222 down	
Rat hemi-section SCI	T9	0, 1, 3, 7, 14, 21 and 28 ​d	RNA-Seq	360DEcircRNAs	Wu et al. [74]
Rat contusion SCI	T10	14 ​d	qRT-PCR	Circ-HIPK3	Zhao et al. [76]

Abbreviations: RNA-Seq: RNA sequencing; T: thoracic level; DEcircRNAs: differentially expressed circRNAs.

### 3.1 The expression profiles of circRNAs in SCI

To investigate the pathophysiology process of the immediate phase of SCI, microarray analysis at 2 ​h after SCI was performed [62]. Liu et al. identified 1101 upregulated and 897 downregulated circRNAs. The dysregulated circRNAs were mainly enriched in neuronal signal transduction and inflammatory response, including neuroactive ligand receptor interaction, long-term potentiation, chemokine and cytokine signaling pathway and so on. From the view of the circRNA-miRNA network, increased expression of circRNA_005470 is considered as the sign of immediate phase.

At the acute phase of SCI (6 ​h post SCI), a total of 150 circRNAs were significantly differentially expressed [63]. Among them, circRNA_07079 and circRNA_01282 could bind miR-351-5p to involve in the pathogenesis of SCI, which depends mainly on the AMP-activated protein kinase (AMPK) signaling pathway and cyclic adenosine monophosphate (cAMP) signaling pathway. Recent studies have proved that activating AMPK signaling pathway exerts neuroprotective effects via promoting neural autophagy and inhibiting neural apoptosis after SCI [64,65]. In addition, increasing the cAMP levels could promote axonal regeneration and myelination, and thus overcome the growth-inhibitory microenvironment [66].

At 3 days after SCI, Qin et al. [67] revealed that differently expressed circRNAs were mainly enriched in carbohydrate metabolic process, AMPK signaling pathway and the peroxisome related pathway. With KEGG analysis, AMPK plays an important role in energy homeostasis, and the isoform abundance of AMPK is determined by physical/neuromuscular activity after SCI [65,68]. After traumatic SCI, peroxisome related pathway triggers the dysregulation of mitochondrial function, but how to exerts the neuroprotective effect needs further research [69,70]. Besides, based on the microarray data of circRNA, miRNA and mRNA from Gene Expression Omnibus (GEO) datasets, we also identified three circRNAs (circRNA_003801, circRNA_014620 and circRNA_013613) and two miRNAs (miR-223-3p and miR-182) in the final circRNA-miRNA-hub gene sub-network, which may play important roles in inflammatory response and nerve regeneration [71]. Interestingly, the dysregulated circRNAs in the mice traumatic SCI models were also identified after 3 days of SCI. On the one hand, Wang et al. [72] found 249 upregulated and 249 downregulated circRNAs, closely associated with cytokine–cytokine receptor interaction, leukocyte migration, cell cycle and phagosome signaling way. On the other hand, another microarray analysis revealed that 909 upregulated circRNAs and 222 downregulated circRNAs were found to be enriched in programmed cell death, inflammatory response and ECM receptor interaction pathway. Among these circRNAs, cicRNA_7079 could attenuate the apoptosis of motor neurons, which may be a potential therapeutic target of SCI [73].

Considering that examination at a single time may ignore the dynamic changes of circRNAs, a comprehensive RNA sequencing analysis at days 0, 1, 3, 7, 14, 21, and 28 post-SCI was performed [74]. This study obtained 360 differentially expressed circRNAs, mainly enriched in cytokine–cytokine receptor interaction and chemotaxis. More importantly, circRNA_01477/miR-423-5p axis may play an essential role in regulating the SCI-induced regeneration microenvironment.

### 3.2 CircRNAs in the pathophysiological process of SCI

**Fig. 1:**
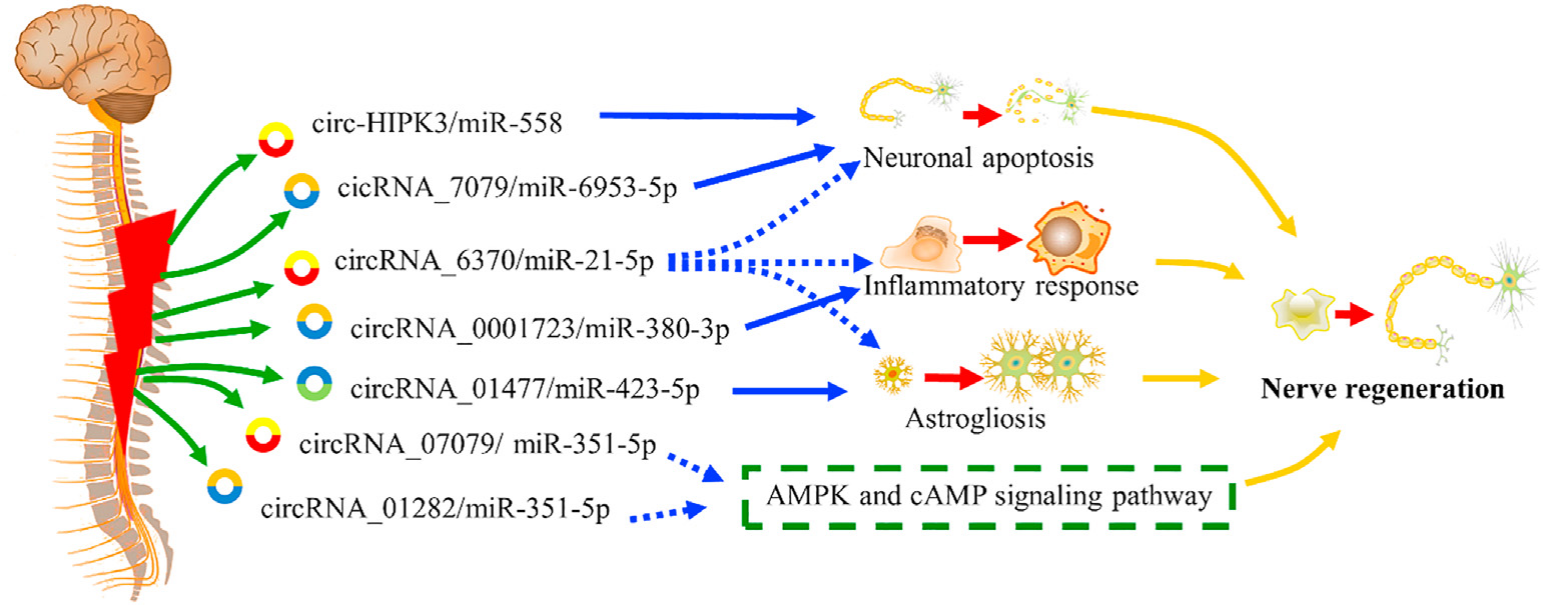
The biological roles and therapeutic potential of circRNAs in SCI.

Notes: solid arrow represents that circRNAs functions were verified by experiments; dotted arrow means circRNAs functions were predicted by bioinformatic analysis.

SCI undergoes primary injury and secondary neurological damage, which involves common pathophysiological mechanisms including blood–brain barrier disruption, inflammation and neuronal apoptosis. Recent studies have indicated that circRNAs play multiple regulatory roles in different pathophysiological processes of SCI (Fig. 1).

The permanent loss of neurons is a major barrier to functional recovery of SCI, so promoting neurons survival and inhibiting neurons apoptosis is essential for the SCI treatment [75]. Zhao et al. [76] has identified the neuroprotective effect of circular RNA homeodomain interacting protein kinase 3 (circ-HIPK3) in SCI rat models. Previous studies have also shown that circ-HIPK3 was connected to neuron autophagy in neurodegenerative diseases [77]. Moreover, the subsequent experiments indicated that circ-HIPK3 could sponge miR-558 to attenuate neuronal cells apoptosis after SCI. To further explore the anti-apoptosis mechanism of circRNAs following SCI, cicRNA_7079 was predicted as a new anti-apoptotic molecule in SCI mice through an apoptosis-related ceRNA network. What's more, knockdown of cicRNA_7079 was found to enhance apoptosis of NSC-34 motor neurons cells via downstream mmu-miR-6953-5p [73].

Inflammation is the most common biological process in the imbalanced microenvironment of SCI, which involves the activation of microglia, astrocytes and infiltration of peripheral immune cells [78]. A recent study demonstrated that circRNA_0001723 sponged miR-380-3p to increase the expression of hypoxia inducible factor-1 (HIF-1α) after SCI, ameliorating inflammatory response by modulating nucleotide-binding domain and leucine-rich repeat protein 3 (NLRP3) inflammasome [79]. Wu et al. [74] then proved that knockdown of circRNA_01477 could regulate the SCI microenvironment by inhibiting astrocyte proliferation and migration. Although the role of circRNA_01477/miR-423-5p axis in SCI has not been verified, the protective effect of miR-423-5p in other diseases was clear, such as pulmonary tuberculosis, myocardial infarction and heart failure [[80], [81], [82]]. In addition, circRNA_6370/miR-21-5p axis has been shown to mediate nerve regeneration and functional recovery by regulating astrogliosis, apoptosis and inflammatory responses after SCI through the PI_3_K/Akt/mTOR pathway, which serve as a promising therapeutic target for SCI [72,83].

Particularly, neuroinflammatory response may be an underlying mechanism of neuropathic pain (NP), which is one of the most common complications of SCI. Current research shows that approximately 65–85% of SCI patients suffer from NP, severely decreasing the quality of life of patients [84]. CircAnks1a, a spinal cord-specific and conserved circRNA, could result in central sensitization and pain behavior induced by spinal nerve ligation (SNL), thus becoming a potential therapeutic target for NP. Apart from mediating the transcriptional regulation of VEGFB, circAnks1a also regulates the expression of VEGFB at posttranscriptional level by sponging miR-324-3p, thereby inducing VEGFB upregulation and NP [85].

To sum up, the evidence demonstrates that circRNAs could exert their effect on neuronal apoptosis, neuroinflammation and astrogliosis after SCI. Considering the multiple roles of circRNAs, manipulating the expression of circRNAs may provide new therapeutic strategies for SCI in the future.

## 4 Conclusion

Spinal cord injury is a major cause of morbidity and mortality worldwide, resulting in permanent neurological impairments and heavy social and economic burden. However, the accurately pathogenic mechanism and promising therapeutic targets remain unclear. Intriguingly, circRNAs exhibit high abundance in neural tissues with regulating neuronal development and synaptic plasticity. Besides, circRNAs could act as miRNAs sponges, regulate gene transcription, and even could be translated into proteins. In the current review, we summarized the recent studies about circRNAs in SCI, which will facilitate a better understanding of the pathogenesis of SCI and promote circRNA-based clinical applications.

Although circRNAs may be a promising clinical biomarkers and treatment targets of SCI in the future, some major problems remain to be solved. First of all, it's important to verify whether dysregulated circrRNAs expression profiles are specifically or casually connected with the pathogenesis of SCI. More research using appropriate models and multicenter, large-scale trials are needed in the future. Besides, considering that these circRNAs/miRNAs interaction networks of SCI were mainly identified by combing RNA sequencing technology and bioinformatics prediction software, we need better evidence to confirm the combination and molecular mechanism based on immunoprecipitation, RNA pulldown and so on. Moreover, the single-cell RNA sequencing may provide new insight into regulation mechanisms of circRNAs under SCI at the level of single cells. Furthermore, the role of exosomal circRNAs in the communication between neurons, glial cells and immune cells in other neurological disorders has been uncovered, but it is still unknown in SCI. Given the distinct advantages of exosomes as effective drug carriers, exosome-medicated circRNAs delivery to injured regions may be a potential pharmacological strategy to enhance functional recovery of SCI.

## Declaration of competing interest

The authors have no conflicts of interest relevant to this article.
